# Ignorance and Ill Health

**Published:** 1882-05

**Authors:** 


### IGNORANCE AND ILL HEALTH.

A lady correspondent, for weary months and years an invalid, writes in reference to a sentiment advanced in one of the Journals—that sickness is the result very generally of ignorance or inattention—says :

"I am willing to let it be, that my illness is the result of ignorance, but, I suppose, if the whole were known, it would be seen that some of my ailments are inherited. I was early taught that if I disobeyed the physical law, illness would be the result. My father was a physician, a regular graduate, but died while I was young, and in a few years I had a new one to care for me, consequently I did not have that kind of training which so nervous a child as I ought to have had ; under more favorable circumstances I might have had better health.

"If I had my life to live over again. I should select such occupations as would be most conducive to health, even if it were all drudgery. Inclined to be ambitious, and not having the means to enable me to "take the world easy," I hurried on, by night and day, until I could do so no longer. We often think in youth, if we could accomplish a desired object, our happiness would be complete, but when attained we are not happy, and sigh for something else. The only thing I sigh for now is good health, and if ever I come into possession of so great a happiness, it will be highly prized."

Such, reader, is the experience of multitudes, learned at a time of life too late for remedy ; have a care then that such may not be yours; let the promotion of your health be a prominent ingredient in every plan and every avocation, and you will feel thankful for the result to the latest hour of your life, for it bears a value far beyond that of glory or of gold.

### RAGE AND RUIN.

Some one has said, that every furious burst of passion shortens a man's life a year. If it only shortened his own life, the world would not be a great loser; but unfortunately, passionate people keep all around them in hot water; their very presence, without a word being said, generates an evil atmosphere.

